# Discussions of Life Expectancy and Changes in Illness Understanding in Patients With Advanced Cancer

**DOI:** 10.1200/JCO.2015.63.6696

**Published:** 2016-05-23

**Authors:** Andrew S. Epstein, Holly G. Prigerson, Eileen M. O’Reilly, Paul K. Maciejewski

**Affiliations:** Andrew S. Epstein, Eileen M. O’Reilly, Memorial Sloan Kettering Cancer Center; Holly G. Prigerson, Paul K. Maciejewski, Center for Research on End-of-Life Care, Cornell University; and Weill Cornell Medicine, New York, NY

## Abstract

**Purpose:**

Accurate illness understanding enables patients to make informed decisions. Evidence of the influence of prognostic discussions on the accuracy of illness understanding by patients would demonstrate the value of discussions.

**Methods:**

Recent and past oncology provider-patient discussions about prognosis/life expectancy were examined for their association with changes in illness understanding by patients. Patients (N = 178) with advanced cancers refractory to prior chemotherapy whom oncologists expected to die within 6 months were interviewed before and after a visit in which cancer restaging scan results were discussed. Illness understanding scores were the sum of four indicator variables: patient terminal illness acknowledgment, recognition of incurable disease status, knowledge of the advanced stage of the disease, and expectation to live months as opposed to years.

**Results:**

Before the restaging scan visit, nine (5%) of 178 patients had completely accurate illness understanding (ie, correctly answered each of the four illness understanding questions). Eighteen patients (10%) reported only recent discussions of prognosis/life expectancy with their oncologists; 68 (38%) reported only past discussions; 24 (13%) reported both recent and past discussions; and 68 (38%) reported that they never had discussions of prognosis/life expectancy with their oncologists. After adjustment for potential confounders (ie, education and race/ethnicity), analysis identified significant, positive changes in illness understanding scores for patients in groups that reported recent only (least-squares mean change score, 0.62; 95% CI, 0.23 to 1.01; *P* = .002) and both recent and past (least-squares mean change score, 0.37; 95% CI, 0.04 to 0.70; *P* = 0.028) discussions of prognosis/life expectancy with their oncologists.

**Conclusion:**

Patients with advanced cancer who report recent discussions of prognosis/life expectancy with their oncologists come to have a better understanding of the terminal nature of their illnesses.

## INTRODUCTION

Informed consent remains a cornerstone of medical decision making, but, for myriad reasons,^1^ oncologists often struggle with whether and how best to convey sensitive information about prognoses to their patients with advanced cancer. Sometimes, oncologists do not share information with patients about a terminal cancer prognosis.^2,3^ However, information from physicians about end-of-life care is often wanted by patients^4,5^ and is related to patient receipt of higher-quality care near death.^3,6^ As highlighted in a recent Institute of Medicine report,^7^ patient-physician communication at the end of life is a promising target for improvement of the delivery of patient-centered end-of-life care.

Often, patients with common advanced cancers have inaccurate illness understanding and would benefit from effective prognostic communication. Many patients mistakenly believe that their cancers are curable when they are not. A recent study of patients with advanced lung and colorectal cancer found that 69% and 81%, respectively, believed that the chemotherapy they received was intended to cure them.^8^ These data suggest a need for oncologists to share prognostic information that is relevant to the understanding of the intent of treatment. Nevertheless, many oncologists are reluctant to do so, because they worry that, by sharing prognostic information, they will make patients needlessly hopeless or upset^3^ and/or that patients will view them less favorably as a result.^8^ Some evidence suggests that, at least in the short run, physicians who share prognostic information are viewed less positively by their patients,^8^ but other findings suggest that the sharing of prognostic information does not damage the oncologist-patient relationship.^4,6^

Research has shown that accurate prognostic understanding is associated with anxiety and worse quality of life^9^ and that training clinicians to communicate about end-of-life care may actually result in higher patient depression scores.^10^ However, patients who report engagement in end-of-life discussions have not been shown to be more depressed or worried.^3,4,6^ Studies show that patients with serious illness do not lose hope,^11^ suffer,^12^ or die sooner^13^ as a result of end-of-life discussions. Bereaved caregivers also do not incur lasting psychological harms from such discussions.^3^ Thus, the effects of prognostic communication on care,^3,6^ patient mood, and patient relationships with their oncologists have been studied. However, research is needed to determine the effect of prognostic communication on illness understanding by patients. Specifically, the impact of prognostic discussions on patient understanding of disease status, curability, and life expectancy has not been examined with data designed explicitly for this purpose. Such data would provide guidance on how to communicate more effectively to promote illness understanding in patients.

This study sought to evaluate the effects of recent and past clinical discussions about prognosis on changes in illness understanding by patients with advanced cancer. We hypothesized that recent and ongoing prognostic discussions would improve illness understanding in patients.

## METHODS

### Study Sample

The analyzed patient sample (N = 178) was drawn from the Coping with Cancer II (CwC-II) study. CwC-II is a National Cancer Institute–funded, prospective, multi-institutional cohort study of patients with advanced cancer and their oncology providers designed to evaluate how end-of-life communication affects illness understanding by patients. Participants were recruited at nine US cancer centers: Dana-Farber/Harvard Cancer Center (DF/HCC; Dana-Farber Cancer Institute, Brigham and Women’s Hospital, and Massachusetts General Hospital, Boston, MA), Parkland Hospital (Dallas, TX), Simmons Comprehensive Cancer Center (Dallas, TX), Yale Cancer Center (New Haven, CT), Meyer Cancer Center at Weill Cornell Medical College (New York, NY), Memorial Sloan Kettering Cancer Center (New York, NY), Virginia Commonwealth University Massey Cancer Center (Richmond, VA), University of New Mexico Cancer Center (Albuquerque, NM), and Pomona Valley Hospital Medical Center (Pomona, CA).

Patients had to meet the following eligibility criteria: stage IV gastrointestinal, lung, or gynecologic cancer and select incurable and poor-prognosis stage III cancers (eg, pancreas and lung); oncologist-estimated life expectancy of 6 or fewer months; disease progression after at least one chemotherapy regimen or, in the case of advanced colorectal cancers, progression during treatment with two chemotherapy regimens. Additional eligibility criteria included age of at least 21 years and the ability to complete the study interviews. Patients with cognitive impairment (eg, rater-perceived inability to provide reliable responses and validly respond to the questions posed of them) were excluded. Institutional review boards of all participating institutions approved study procedures, and all participants provided written, informed consent.

This study focused on changes in illness understanding by patients before and after a visit with his or her oncology provider to discuss scan results and to evaluate disease progression. A total of 178 CwC-II participants who completed both pre- and post-scan visit interviews between January 2011 and February 2015 were included in the analyses.

### Measures

#### Patient characteristics.

Patients provided information about age, sex, race/ethnicity, education, marital status, and health insurance status.

#### Changes in illness understanding by patients.

In pre- and post-scan interviews, patients were asked four questions that assessed their terminal illness acknowledgment (TIA), recognition of their incurable disease status, knowledge of the advanced stage of their disease, and expectation to live months as opposed to years. These elements of illness understanding were deemed by us to be essential for patients to make informed decisions about end-of-life care. Responses were coded 1 or 0 to indicate the presence or absence, respectively, of each of these elements of illness understanding by patients. These four indicators were then added together to construct summary scores (possible range, 0 to 4) to reflect illness understanding at the times of both the pre- and post-scan visit interviews. Differences between pre- and post-scan visit illness understanding scores (possible range, −4 to 4) were used to define changes in illness understanding by a patient between te pre- and post-scan visit interviews.

TIA was assessed with the question “How would you describe your current health status?” Response options were (1) relatively healthy, (2) relatively healthy and terminally ill, (3) seriously ill but not terminally ill, (4) seriously ill and terminally ill, and (5) do not know. TIA was coded 1 for responses options 2 and 4 and 0 for response options 1, 3, and 5.

Recognition of an incurable disease status was assessed with the question “Which of the following best represents what your oncology providers have told you about a cure for your cancer?” Response options were (1) my cancer will be cured, (2) my cancer may be cured if treatments are successful, (3) my cancer cannot be cured but we will try to control the cancer with treatment, (4) my cancer cannot be cured and I am not able to have any additional cancer treatment, and (5) do not know. Recognition of incurable disease was coded 1 for response options 3 and 4 and 0 for response options 1, 2, and 5.

Knowledge of advanced stage of cancer was assessed with the question “What stage is your cancer?” Responses were (1) no evidence of cancer, (2) early stage of cancer, (3) middle stage of cancer, (4) late stage of cancer, (5) end stage of cancer, and (6) do not know. Knowledge of advanced stage of cancer was coded 1 for responses options 4 and 5 and 0 for response options 1, 2, 3, and 6.

Expectation to live months as opposed to years was assessed with the question “Many patients have thoughts about how having cancer might affect their life expectancy, either on the basis of what their doctors have told them, what they have read, or just their own sense about how long they might live with cancer. When you think about this, do you think in terms of (select response)?” Response options were (1) months, (2) years, and (3) do not know. Expectation to live months as opposed to years was coded 1 for response option 1 and 0 for response options 2 and 3.

#### Patient-oncologist discussions of prognosis/life expectancy.

During the post-scan visit, patients were asked “At the last oncology visit, was there any discussion of your prognosis or life expectancy with this disease?” and “Have you discussed your prognosis/life expectancy with your oncology provider in past visits?” Response options for each of these questions were yes or no. Responses to these questions were used to identify patients who reported discussions at only recent (last visit), only past (prior to last visit), or both recent and past visits or never having discussions of prognosis/life expectancy with the oncologist.

### Statistical Analysis

To evaluate potential sociodemographic confounders, differences in age and education between groups of patients who reported recent only, past only, both recent and past, and never having discussions of prognosis/life expectancy with oncologists were examined with means and standard deviations (SDs); between-group differences in sex, race/ethnicity, marital status, and insurance status were examined with distributions of absolute and relative frequencies. Correlations between age or education and pre- and post-scan visit illness understanding scores, and change in illness understanding score, were used to evaluate associations between age or education and illness understanding. Change in illness understanding and differences in pre-scan visit and post-scan visit understanding on the basis of sex, race/ethnicity, marital status, and insurance status were examined with means and standard deviations.

Mean changes in illness understanding scores for patients who reported recent only, past only, both recent and past, and never having discussions of prognosis/life expectancy with oncologists, adjusted for potential patient demographic confounders, were estimated via least-squares means in a generalized linear model of changes in illness understanding by patients. Minimum effect sizes for changes in illness understanding scores for tests with adequate (80%) statistical power for the full sample (N = 178) and for subgroups with 20 or 60 patients were 0.17, 0.53, and 0.29, respectively. Time between interviews was unrelated to changes in illness understanding and, therefore, was not considered a confounder in the analysis.

Statistical analysis was conducted with SAS statistical software, version 9.4 (Cary, NC) and was based on two-sided tests. *P* < .05 was considered statistically significant.

## RESULTS

The median time between the pre- and post-scan interviews was 6 weeks (range, 1 to 32 weeks). At pre-scan interviews, 32 patients (18%) had an illness understanding score of 0; 48 (27%), of 1; 47 (26%), of 2; 42 (24%), of 3; and nine (5%), of 4. Post-scan interviews revealed that 26 (15%), 46 (26%), 49 (28%), 44 (25%), and 13 (7%) had illness understanding scores of 0, 1, 2, 3, and 4, respectively. Between interviews, three patients (2%) had a change in illness understanding score of −2; 29 (16%) had a change score of −1; 96 (54%) had a change score of 0; 41 (23%) had a change score of +1; and nine (5%) had a change score of +2. According to patient reports, 18 (10%) had only recent discussions of prognosis/life expectancy with the oncologist; 68 (38%) had only past discussions; 24 (13%) had both recent and past discussions; and 68 (38%) never had discussions of prognosis/life expectancy with the oncologist.

As listed in Table 1, patients who reported only past (n = 68) or both recent and past (n = 24) discussions were more highly educated (mean years of education: 15.3; SD, 3.0 and 15.3; SD, 2.7, respectively) than patients who reported only recent (n = 18) or never having (n = 68) discussions (mean years of education: 13.4; SD, 2.9 and 13.3; SD, 4.2, respectively). Black patients were more highly represented in the recent only and never having discussions groups (4 [22%] of 18 patients and 15 [22%] of 68 patients, respectively) and were less highly represented in the past discussions only group (3[4%] of 68 patients). Married patients were more highly represented in the only past discussions group (47 [70%] of 67 patients) and were less represented in the both recent and past discussions group (10 [43%] of 23 patients).

**Table 1.** Patient Characteristics and Their Associations With Reported Discussions of Prognosis/Life Expectancy

Patient Characteristic	Full Sample (N = 178)		Reported Discussions of Prognosis/Life Expectancy							
			Recent Only (n = 18; 10.1%)		Past Only (n = 68; 38.2%)		Both (n = 24; 13.5%)		Never (n = 68; 38.2%)	
	No.	%	No.	%	No.	%	No.	%	No.	%
Age, years, mean (SD)	59.7 (9.9)		57.3 (12.2)		60.3 (9.8)		58.5 (7.9)		60.1 (10.0)	
Education, years, mean (SD)	14.4 (3.6)		13.4 (2.9)		15.3 (3.0)		15.3 (2.7)		13.3 (4.2)	
Sex										
Male	58	32.8	6	33.3	15	22.4	9	37.5	28	41.2
Female	119	67.2	12	66.7	52	77.6	15	62.5	40	58.8
Race/ethnicity										
Black	25	14.0	4	22.2	3	4.4	3	12.5	15	22.1
Latino	19	10.7	1	5.6	6	8.8	3	12.5	9	13.2
White	134	75.3	13	72.2	59	86.8	18	75.0	44	64.7
Marital status										
Married	101	57.7	10	55.6	47	70.1	10	43.5	34	50.7
Not married	74	42.3	8	44.4	20	29.9	13	56.5	33	49.3
Insurance status										
Insured	149	83.7	14	77.8	62	91.2	21	87.5	52	76.5
Not insured	29	16.3	4	22.2	6	8.8	3	12.5	16	23.5

NOTE: Missing data: age (n = 1), sex (n = 1), marital status (n = 3).

Abbreviation: SD, standard deviation.

Table 2 lists changes in illness understanding scores by characteristic, including the following: Black patients (n = 25) had negative changes in illness understanding scores (mean, −0.24; SD, 1.01), and Latino (n = 19) and white (n = 134) patients had positive changes in illness understanding scores (mean, 0.37; SD, 0.76 and mean, 0.17; SD, 0.75, respectively). Overall, patients (N = 178) had positive changes in illness understanding scores (mean, 0.13; SD, 0.81).

**Table 2.** Associations Between Patient Characteristics and Illness Understanding Scores

Patient Characteristic	No. of Patients	Illness Understanding Score								
		Pre-Scan Visit			Post-Scan Visit			Change		
		Mean	SD	*r*	Mean	SD	*r*	Mean	SD	*r*
Overall	178	1.71	1.16		1.84	1.17		0.13	0.81	
Age, years	177			0.00			0.04			0.05
Education, years	178			0.14			0.24			0.14
Sex										
Male	58	1.52	1.13		1.62	1.17		0.10	0.83	
Female	119	1.78	1.16		1.94	1.16		0.16	0.79	
Race/ethnicity										
Black	25	1.72	1.10		1.48	0.92		−0.24	1.01	
Latino	19	1.63	1.30		2.00	1.05		0.37	0.76	
White	134	1.72	1.16		1.89	1.22		0.17	0.75	
Marital status										
Married	101	1.67	1.23		1.82	1.15		0.15	0.73	
Not married	74	1.74	1.07		1.88	1.20		0.14	0.91	
Insurance status										
Insured	149	1.68	1.14		1.81	1.21		0.13	0.82	
Not insured	29	1.86	1.27		2.03	0.94		0.17	0.76	

NOTE: Missing data: age (n = 1), sex (n = 1), marital status (n = 3). *r* indicates the correlation coefficient.

Abbreviation: SD, standard deviation.

Table 3 lists mean changes in elements of illness understanding, stratified by discussions of prognosis/life expectancy. The largest contributors to changes in illness understanding among patients who reported only recent discussions of prognosis/life expectancy (n = 18; mean change, 0.50; SD, 0.86) were changes in understanding of incurability (mean, 0.17; SD, 0.38) and late stage of disease (mean, 0.17; SD, 0.62). The largest contributors to changes in illness understanding among patients who reported both recent and past discussions (n = 24; mean change, 0.38; SD, 0.92) were changes in understanding of late stage of disease (mean, 0.21; SD, 0.51) and in expectations to live months, not years (mean, 0.21; SD, 0.41).

**Table 3.** Composition of Change in Illness Understanding Score, Stratified by Discussions of Prognosis/Life Expectancy

Reported Discussions of Prognosis/Life Expectancy	No. of Patients	Mean Changes in Elements of Illness Understanding								Total Score*	
		TIA		Incurable		Late Stage		Months to Live			
		Mean	SD	Mean	SD	Mean	SD	Mean	SD	Mean	SD
Only recent	18	0.11	0.58	0.17	0.38	0.17	0.62	0.06	0.42	0.50	0.86
Only past	68	0.00	0.42	0.12	0.37	0.09	0.38	−0.04	0.27	0.16	0.64
Both recent and past	24	−0.13	0.45	0.08	0.50	0.21	0.51	0.21	0.41	0.38	0.92
Never	68	−0.07	0.50	0.00	0.39	0.03	0.46	−0.03	0.17	−0.07	0.85

NOTE. Changes refer to difference between pre- and post-scan illness understanding.

Abbreviations: SD, standard deviation; TIA, terminal illness acknowledgment.

* Total score is the summation of the four elements of illness understanding (ie, TIA, incurable, late state, months to live).

In Table 4, results adjusted for potential confounders (ie, patient years of education and race/ethnicity) are listed. Groups of patients who reported recent only and both recent and past discussions of prognosis/life expectancy with their oncologists had significant, positive changes in their illness understanding scores (least-squares mean change score: 0.62; 95% CI ,0.23 to 1.01; *P* = .002 and 0.37; 95% CI, 0.04 to 0.70; *P* = .028, respectively).

**Table 4.** Changes in Patient Illness Understanding in Relation to Discussions of Prognosis/Life Expectancy

Discussion of Prognosis/Life Expectancy	No. of Patients	Change in Illness Understanding Score		*P*
		LS Mean	95% CI	
Only recent	18	0.62	0.23 to 1.01	.002
Only past	68	0.15	−0.09 to 0.39	.221
Both recent and past	24	0.37	0.04 to 0.70	.028
Never	68	0.02	−0.21 to 0.24	.885

Abbreviation: LS, least-squares mean score adjusted for patient race/ethnicity and years of education.

## DISCUSSION

Results of this study demonstrate how poorly patients with advanced cancer understand their prognoses and how effective recent prognostic discussions are to improve illness understanding by patients. All enrolled patients in this study had incurable cancer that was at an advanced stage (eg, late, stage IV gastrointestinal cancer) and a life expectancy of months, not years. A small minority of patients accurately, and completely, understood the gravity of their illnesses (eg, 5% endorsed each element of the terminal prognosis at study entry); approximately one in four (23%) reported only recent or recent and past discussion of prognosis with the oncologist. Patients who reported at least a recent discussion about prognosis with the oncology provider exhibited significant improvements in illness understanding. These results highlight the need for timely (ie, current) prognostic disclosures to terminally ill patients who meet the criteria used for this study. The results also suggest that oncologists should discuss prognosis on an ongoing basis, and as frequently as appropriate, with their terminally ill patients. If this occurred, patients would likely have better illness understanding and, thus, make more informed decisions about their end-of-life care.

These results are consistent with, and advance, the existing literature on illness understanding, prognostic disclosure, and advance care planning. The effect of recently reported prognostic discussions on improvements in illness understanding by patients is in line with the advance care planning strategy to regularly and dually address both dynamic medical situations and individual patient goals. This approach encourages medical decisions to be made in the moment^14^ instead of on the basis of advance directive documents, which can sometimes be nonspecific, outdated, or unavailable.^15,16^ Consideration of prognostic understanding as an evolving awareness of one’s changing health empowers patients, their loved ones, and their healthcare team to make informed decisions. Furthermore, recognition of the need to update patients frequently about a prognosis may help patients and families who struggle^17^ to come to terms with the terminal nature of a disease.

In the delicate task of delivering prognoses, some have argued that the median informs the message,^18^ which argues for the use of a prognostic range such as months instead of communication of a specific time frame, such as 6 months to live. Outcomes research in strategies of communicating with both realism and hope^18^ for patients with serious illness is needed; statements, such as hoping for the best (eg, years of survival) while being prepared for the worst (eg, months left to live), during ongoing discussions of prognosis may be one way to achieve a balance. This report suggests that, regardless of the approach, the recency of the prognostic discussion matters for prognostic understanding by the patient. Future research is needed to identify the most effective ways to communicate prognostic information to ensure that patients have accurate illness understanding. Such insight seems to be a prerequisite for informed decision making.

There are strengths and limitations to our study. One strength is that our data were drawn from a large, prospective, observational cohort of patients from several centers and with cancers representative of common terminal illnesses, in a study explicitly designed to discern the effects of oncology provider communication on terminal illness understanding by patients. To our knowledge, our study is the first to directly address and demonstrate these associations between the timing of patient-reported prognostic discussions and improvements in illness understanding by patients. One weakness is that patients who do not know their cancer stages or prognoses might also inaccurately recall whether their doctors have talked with them about such topics. This notion of discord, because of optimism bias^19^ or misunderstanding between what physicians say and what patients^19^ or caregivers^20^ hear has been described in oncology settings.^20^ If patients misheard what was said, the effect was likely in the direction of underreporting prognostic discussions; this would suggest that prognostic discussions by oncologists would have less impact. We contend that, for informed decision making, how patients hear and understand what their oncology providers say about their illnesses matters the most.

Despite these limitations, future research directions include additional elucidation of the communication elements that are beneficial (or deleterious) to patient understanding and that promote advance care planning. Studies to investigate the combined use of booklets and audio recordings^21^ for education to patients with cancer about the chemotherapies they will receive have proven effective. Larger-scale validation of these approaches is warranted, as is ongoing research into other educational media, such as videos,^22^ for these and other topics of importance to patients with cancer and their loved ones. Communication decision aids for caregivers themselves have also been effective advance care planning strategies.^23^ Although the impact of communication skills training on patient outcomes has recently been called into question,^10^ other data show that communication skills training, whether as workshops^24-26^ or technologies for individual practice,^27^ can at least help clinicians acquire these important communication skills. Ongoing research into communication skills training needs to examine the interplay between cognitive information delivery (eg, communication about the prognostic realities of an advanced cancer as realistically and hopefully as possible) and response to emotion (eg, patient sadness, anxiety, anger) with empathic responses and the effects that such skills have on illness understanding by patients.^28^

In conclusion, patients with advanced cancer who acknowledge recent or ongoing discussions of prognosis/life expectancy with their oncology providers come to have a better understanding of the terminal nature of their illnesses and, thus, may be better prepared to make informed end-of-life care decisions.
